# The Historical Evolution of Head and Neck Tumor Pathology in WHO Classifications: From Morphology to Molecular Taxonomy

**DOI:** 10.1007/s12105-026-01962-8

**Published:** 2026-10-26

**Authors:** Fernanda Viviane Mariano, Luccas Lavareze, Arthur Antolini-Tavares, Alessandro Franchi, Alfons Nadal, Roderick Simpson, Ilmo Leivo, Martina Bradová, Alena Skálová, Reydson Alcides de Lima-Souza, Alfio Ferlito

**Affiliations:** 1 Department of Pathology, School of Medical Sciences, University of Campinas (UNICAMP), Rua Tessália Vieira de Camargo, 126 - Cidade Universitária, Campinas, SP 13083-887 Brazil; 2 Oral Diagnosis Department, Piracicaba Dental School, Universidade Estadual de Campinas (FOP-UNICAMP), Piracicaba, SP Brazil; 3 Section of Pathology, Department of Translational Research, University of Pisa, Pisa, Italy; 4 Department of Pathology, Hospital Clinic, Barcelona, Spain; 5 Department of Basic Clinical Practice, School of Medicine, Universitat de Barcelona, Barcelona, Spain; 6 Department of Pathology, University of Calgary, Calgary, AB Canada; 7 Institute of Biomedicine, Pathology, Department of Pathology, University of Turku, Turku University Hospital, Turku, Finland; 8 Bioptic Laboratory, Ltd, Pilsen, Czech Republic; 9 Department of Pathology, Faculty of Medicine in Pilsen, Charles University, Pilsen, Czech Republic; 10 Coordinator of the International Head and Neck Scientific Group, Padua, Italy

**Keywords:** Head and neck pathology, World Health Organization, Tumor taxonomy, Histopathology, Molecular pathology, Precision oncology

## Abstract

**Purpose:**

The WHO classification of head and neck tumors over the past five decades were reviewed, highlighting the major conceptual and taxonomic changes that have shaped contemporary head and neck pathology.

**Methods:**

Major WHO classifications of head and neck tumors were reviewed, with a focus on changes in diagnostic concepts and taxonomic principles rather than individual additions or removals of tumor entities.

**Results:**

WHO classifications have evolved from morphology-based, site-specific systems to an integrated clinicopathological and molecular taxonomy. Early editions emphasized histomorphology and presumed tissue of origin. Subsequent classifications, however, increasingly incorporated immunophenotypic and molecular findings. The recognition of virus-associated neoplasms and recurrent molecular alterations has contributed to defining new entities and refining existing categories. The fifth edition further standardized tumor taxonomy by introducing essential and desirable diagnostic criteria, reflecting a biology-driven approach to classification.

**Conclusion:**

The evolution of WHO classifications reflects major advances in tumor biology and has substantially influenced diagnostic practice and clinical interpretation. Future classifications will likely incorporate increasingly sophisticated molecular, digital, and computational approaches while maintaining standardized, reproducible, and clinically meaningful diagnostic criteria.

## Introduction

The classification of head and neck tumors has undergone remarkable transformations over the past five decades. Since the publication of the first World Health Organization (WHO) histological typing manuals in the early 1970s, advances in pathology, molecular biology, epidemiology, and oncology have progressively reshaped the definition and categorization of these tumors. The WHO Classification of Tumors has played a central role in this process by providing an international framework for diagnosis, research, and patient management [1–6].

Successive editions have reflected major advances in tumor biology, progressively incorporating immunohistochemistry, virus-associated carcinogenesis, molecular alterations, and genomic biomarkers into tumor classification. These developments have broadened the scope of classification, offering a more comprehensive framework for defining biologically and clinically significant disease entities [7–9].

This evolution has also been accompanied by changes in how the classification process is organized. The early fascicles were developed by a relatively small number of experts [1–3], subsequent editions incorporated a broader range of authors, larger editorial boards, and multidisciplinary expertise. They also underwent extensive peer review and increased participation from clinicians and radiologists, which strengthened the classification’s clinical relevance and scientific robustness [7–9].

This review examines the historical development of the WHO classification of head and neck tumors, from the early fascicle era to the fifth edition. Instead of focusing on the addition or removal of individual entities, this review highlights the major conceptual changes that have shaped modern tumor taxonomy. These changes include the transition from morphology to molecular pathology, the recognition of etiologically defined diseases, and the establishment of a standardized taxonomic framework across head and neck pathology.

## The Evolution of the WHO Classification of Head and Neck Tumors

The WHO classification of head and neck tumors emerged from a simple but decisive need: to give pathologists a shared diagnostic language. Before the WHO introduced its histological typing fascicles, tumor nomenclature often varied among institutions and geographic regions. This limited the comparability of clinicopathological studies, epidemiological data, and treatment outcomes. The earliest classifications were designed to provide reproducible diagnostic criteria based on the best pathological knowledge available at the time [1–3, 5, 6]. However, over subsequent decades, advances in molecular pathology have transformed the classification system. The system evolved from a largely descriptive morphological framework into an integrated taxonomy that incorporates immunophenotypic, molecular, etiologic, and clinical features [7–9].

## 1971–1978 - The Fascicle Era: Building a Common Diagnostic Vocabulary

During the 1970s, the WHO adopted a different approach to head and neck pathology, presenting it in a series of site-specific fascicles rather than in a single, integrated classification. Separate volumes were devoted to oral and oropharyngeal tumors, odontogenic tumors and jaw lesions, salivary gland tumors, and tumors of the upper respiratory tract. Though these publications were relatively simple by modern standards, they established the first internationally standardized framework for diagnosing head and neck neoplasms. The primary objective was to improve consistency in terminology and facilitate comparisons among institutions, countries, and clinical studies [2].

The early fascicles were largely morphology-based and reflected contemporary concepts of histogenesis. Tumors were classified according to architectural and cytologic features observed in routine hematoxylin and eosin sections, with little contribution from ancillary techniques. Broad categories such as epithelial tumors, mesenchymal tumors, melanocytic lesions, tumors of uncertain histogenesis, and tumor-like conditions were commonly used. The 1971 classification, for example, explicitly separated true neoplasms from tumor-like conditions. This included lesions such as pyogenic granuloma and mucocele. This illustrates the early effort to establish clearer boundaries between neoplastic and reactive processes [2].

Of the various head and neck subspecialties, odontogenic and salivary gland pathology posed challenges due to their remarkable morphological diversity. Early odontogenic classification, shaped mainly by Jens J. Pindborg and Ivor R. H. Kramer, attempted to organize lesions derived from tooth-forming tissues; however, the distinction between epithelial, ectomesenchymal, and mixed tumors remained imperfect. Additionally, some fibro-osseous lesions were still grouped with odontogenic entities because of their occurrence in the jaws and their periodontal associations, illustrating the difficulty of reconciling anatomical location with presumed tissue of origin [10].

Similarly, the 1972 salivary gland fascicle, prepared by A. C. Thackray with Leslie H. Sobin and international collaborators, addressed one of the most heterogeneous groups in surgical pathology, displaying multiple lines of differentiation within individual neoplasms. Even at this early stage, it became evident that morphology alone was limited, as some tumors exhibited biological behavior that their microscopic appearance could not fully predict (e.g. acinic cell and mucoepidermoid carcinoma, previously described as mucoepidermoid and acinic cell tumor). These early observations contributed to the subsequent recognition that tumor classification required consideration of biological behavior in addition to morphology, laying the groundwork for the later incorporation of immunophenotypic, molecular, and etiologic information into WHO classifications [3].

Neuroendocrine neoplasms are an early example of the limitations of the classification system at that time. In the 1978 classification of upper respiratory tract tumors, neuroendocrine lesions were represented by a broad morphological framework, reflecting the limited understanding of their biological and histogenetic diversity [11]. Subsequent literature increasingly recognized distinct forms of laryngeal neuroendocrine neoplasia with markedly different clinical behaviors, paving the way for their more precise separation in subsequent WHO editions [12, 13]. 

## 1991–1997 - Refining Morphology: Histogenesis, Behavior, and Clinical Risk

Throughout the 1990s, the second generation of WHO fascicles was published. This generation focused less on expanding the number of recognized entities and more on refining morphologic criteria, histogenetic concepts, and clinicopathologic correlations. Although the classification system remained fundamentally morphology-based, there was an increased emphasis on biological behavior, prognostic stratification, and the recognition that tumors with similar microscopic appearances could have different clinical outcomes. These developments demonstrate the gradual shift toward a more clinically relevant diagnostic framework [5, 6, 14–16].

Several anatomical sites underwent significant revisions. The 1991 classification of tumors of the upper respiratory tract and ear, under K. Shanmugaratnam and L. H. Sobin’s supervision, broadened the scope of head and neck pathology by incorporating lesions of the nasal passages, nasopharynx, larynx, hypopharynx, trachea, and ear into a more comprehensive diagnostic system [15, 16]. Neuroendocrine neoplasms exemplify the refinements achieved during this period particularly well. The 1991 classification added atypical carcinoid tumor and small cell neuroendocrine carcinoma to the previously recognized carcinoid tumor and paraganglioma categories . This reflected a more detailed recognition of the morphological and biological spectrum of laryngeal neuroendocrine neoplasms. This distinction was clinically relevant because these tumors exhibited substantially different behaviors: atypical carcinoid tumors had greater metastatic potential, while small cell neuroendocrine carcinoma was highly aggressive. Thus, the classification moved beyond merely recognizing neuroendocrine differentiation to distinguishing entities based on differentiation and biological behavior [17, 18]. This broader anatomical organization provided a more unified framework for classifying tumors that arise in anatomically contiguous regions of the upper aerodigestive tract and related structures.

Meanwhile, salivary gland pathology moved beyond broad descriptive categories. Previously, heterogeneous groups were subdivided into more reproducible entities. For example, the former “monomorphic adenomas” category was separated into more specific entities, including oncocytoma, myoepithelioma, canalicular adenoma, ductal papillomas, and cystadenomas [6]. In malignant tumors, mucoepidermoid and acinic cell tumors were consolidated as carcinomas. The 1991 classification system recognized significant differences in the clinical behavior and treatment responses of salivary carcinomas. Some entities, including polymorphous adenocarcinoma, formerly termed polymorphous low-grade adenocarcinoma, and papillary cystadenocarcinoma, were considered to have a more favorable prognosis, illustrating the evolving recognition of prognostic heterogeneity among salivary gland carcinomas. Papillary cystadenocarcinoma is currently classified within the salivary carcinoma, NOS spectrum [6]. The growing focus on prognostic heterogeneity underscored the limitations of morphology alone and emphasized the need for more precise subclassification and grading systems. These concepts would become increasingly important in subsequent editions [5, 14].

The classification of odontogenic pathology also underwent substantial refinement. The 1992 classification system, organized by Kramer, Pindborg, and Mervyn Shear, was based on their presumed tissue of origin and ability to produce dental hard tissues. This approach provided a more systematic way to distinguish between epithelial, ectomesenchymal, and mixed odontogenic tumors. It also made it easier to distinguish fibro-osseous and bone lesions from true odontogenic neoplasms [5, 19].

Another conceptual advance occurred in 1997 with the publication of the classification of oral cancer and precancer produced by Pindborg, Reichart, Smith, and van der Waal. Unlike previous volumes, this work incorporated clinical risk assessment into the classification framework. It addressed clinically defined disorders, such as leukoplakia and erythroplakia, as conditions associated with an increased risk of malignant transformation. However, it maintained oral epithelial dysplasia as the primary histopathological indicator of risk for malignant transformation [20]. 

Together, these developments reflected a growing recognition that classifications should describe tumor morphology and provide meaningful information about biological behavior and cancer risk [20].

## 2005 - Integration and Genetics: The First Unified Head and Neck Blue Book

A major transition occurred in 2005 with the publication of Pathology and Genetics of Head and Neck Tumors, edited by Leon Barnes, John W. Eveson, Peter Reichart, and David Sidransky. For the first time, the WHO replaced its previous series of site-specific fascicles with one comprehensive volume covering the full range of head and neck neoplasms. This structural reorganization represented more than a format change; it reflected a broader conceptual shift toward multidisciplinary tumor characterization. Although morphology remained the basis of diagnosis, more attention was given to immunophenotypic profiles, genetic alterations, clinical presentation, and biological behavior [7].

The incorporation of molecular findings was particularly significant. While molecular alterations were not yet used as diagnostic criteria, recurrent genetic abnormalities were systematically incorporated into discussions of tumor pathogenesis and classification. Thus, this edition marked the transition from traditional morphology-based pathology to the emerging field of molecular oncology. Genetic findings were presented as complementary tools capable of improving diagnostic accuracy and providing insights into tumor development and progression, rather than replacing conventional histopathology [7].

Several chapters underwent significant revisions. In salivary gland pathology, the classification system solidified the recognition of new entities and improved the understanding of tumor progression. The identification of recurrent alterations involving *PLAG1* and *HMGA2* in pleomorphic adenoma [21], as well as *CRTC1/3::MAML2* in mucoepidermoid carcinoma [22, 23], represented a paradigm shift within the 2005 classification system. These findings are classic examples of the growing integration of molecular genetics into head and neck tumor pathology [7].

In this edition, carcinoma ex pleomorphic adenoma was standardized as a model of tumor progression, emphasizing intracapsular, minimally invasive, and frankly invasive forms. Numerous studies have contributed to our broader understanding of carcinoma ex pleomorphic adenoma, particularly by emphasizing the importance of invasion, carcinomatous subtype, and molecular progression when interpreting this condition [7, 24–27].

The 2005 edition of odontogenic pathology was especially controversial. The adenomatoid odontogenic tumor was moved from the mixed odontogenic category to the epithelial odontogenic tumor group because the neoplastic component is essentially epithelial. Calcifications or dentinoid-like material were deemed insufficient evidence of a true ectomesenchymal neoplastic component [5, 28]. More controversially, the odontogenic keratocyst was renamed the keratocystic odontogenic tumor based on its locally aggressive behavior, high recurrence rate, association with nevoid basal cell carcinoma syndrome, and PTCH1 alterations in the Hedgehog pathway [5, 29]. Similarly, calcifying odontogenic cyst was renamed calcifying cystic odontogenic tumor. Additionally, this chapter did not describe the jaw cysts [7].

The 2005 edition also incorporated advances in the understanding of malignancies of the sinuses and nasal cavity, nasopharyngeal carcinoma, neuroendocrine neoplasms, and precursor lesions of the upper aerodigestive tract into a unified framework that emphasized clinicopathologic correlation and biological behavior. Attention was given to lesions associated with viral oncogenesis, especially those related to the Epstein–Barr virus, such as nasopharyngeal carcinoma. Poorly differentiated and undifferentiated carcinomas also received attention, as they remain diagnostically challenging despite increasing immunohistochemical support [7].

Additionally, in this edition, neuroendocrine neoplasms were organized by degree of differentiation and cytomorphologic features. They were classified as typical carcinoid, atypical carcinoid, small cell carcinoma of neuroendocrine type, combined small cell carcinoma, and paraganglioma [7]. The classification also incorporated immunohistochemical and ultrastructural evidence of neuroendocrine differentiation. Moreover, differences in clinical behavior were recognized among these entities, with atypical carcinoid and small cell carcinoma showing substantially more aggressive behavior than typical carcinoid [30–32]. 

By the end of this period, head and neck pathology had entered a transitional phase in which morphology remained essential but molecular and pathogenetic discoveries increasingly influenced the classification and understanding of tumors.

## 2017 - Viruses and Molecular Entities Enter Routine Classification

The fourth edition of the WHO Classification of Head and Neck Tumors, published in 2017 by Adel K. El-Naggar, John K. C. Chan, Jennifer R. Grandis, Takashi Takata, and Pieter J. Slootweg, represented one of the most significant paradigm shifts in the history of head and neck pathology. Building upon the integration of molecular data introduced in the 2005 edition, this edition firmly established viral oncogenesis and molecular alterations as central components of tumor classification. For the first time, classification was not solely based on morphology and presumed histogenesis but increasingly reflected the biological and etiological mechanisms driving tumor development [8].

One of the most significant changes was the formal recognition of HPV-associated and HPV-independent oropharyngeal squamous cell carcinomas as distinct diseases. Accumulating evidence has demonstrated that HPV-driven tumors differ substantially from conventional squamous cell carcinomas in terms of epidemiology, morphology, molecular profile, treatment response, and prognosis. Incorporating p16 immunohistochemistry and HPV testing into routine diagnostic practice fundamentally altered the approach to diagnosing oropharyngeal cancer, representing a landmark moment in the evolution of head and neck tumor classification [33–35].

The fourth edition also corrected some controversial odontogenic decisions from 2005. Keratocystic odontogenic tumor returned to the cyst category as odontogenic keratocyst, because its biological behavior, although aggressive, was not considered sufficient to define it as a true neoplasm in all cases. Calcifying cystic odontogenic tumor also returned to the cyst category, and jaw cysts were reincorporated into the classification. The ameloblastoma classification was simplified: “solid/multicystic ameloblastoma” was replaced by conventional ameloblastoma, with unicystic and peripheral/extraosseous subtypes maintained separately [4, 33, 36].

This edition reflected the growing influence of molecular pathology across multiple anatomical sites. Recurrent genetic alterations were increasingly viewed not just as biological observations, but also as defining features of specific neoplasms. This shift marked the beginning of the era of molecularly defined tumor entities and laid the groundwork for the taxonomy-driven approach adopted in subsequent WHO classifications [8]. One of the clearest examples of this shift was the advancement in salivary gland pathology. Following the discovery of the *ETV6::NTRK3* fusion, secretory carcinoma, formerly known as mammary analogue secretory carcinoma, was incorporated into the classification [37]. This recognition showed how molecular findings could redefine heterogeneous diagnostic categories and create new disease entities [37].

In salivary gland pathology, there was a refinement in the nomenclature, such as polymorphous adenocarcinoma, replacing the older term polymorphous low-grade adenocarcinoma because not all cases behave as strictly low-grade tumors [33]. Cribriform adenocarcinoma of salivary gland origin, described by Michal, Skálová, Simpson, and collaborators, was placed within the polymorphous adenocarcinoma spectrum [38]. Intraductal carcinoma was also recognized [39], helping reorganize lesions previously called low-grade salivary duct carcinoma or low-grade cribriform cystadenocarcinoma. Additionally, in this classification, entities previously considered independent (e.g., cystadenocarcinoma and intestinal-type adenocarcinomas) were incorporated into the adenocarcinoma NOS (not otherwise specified) spectrum. This was done primarily for two reasons: to simplify the diagnostic process and because they lack clinical or biological significance when considered in isolation [8].

In the sinus and nasal cavity pathology, the growing recognition of NUT carcinoma and other poorly differentiated neoplasms characterized by specific molecular alterations has been reported. These developments foreshadowed the significant increase in molecularly defined sinus and nasal tumor entities that occurred in the fifth edition [8].

The 2017 edition further refined the morphological spectrum of head and neck neuroendocrine neoplasms. It distinguished between well-, moderately-, and poorly-differentiated neuroendocrine carcinomas. The poorly-differentiated category was subdivided into small-cell and large-cell types [8]. These categories reflected the growing recognition that morphologically distinct neuroendocrine neoplasms differ substantially in their clinical behavior [40, 41]. However, the terminology remained closely linked to the traditional carcinoid–neuroendocrine carcinoma framework. This framework differs from the emerging WHO/IARC terminology adopted by other organ systems.

## 2022/2024 - From Classification to Taxonomy: Criteria, Selectivity, and Molecular Definition

The fifth edition of the WHO Classification of Head and Neck Tumors, released online in 2022 and published in print in 2024, represents the culmination of a decades-long transition from descriptive morphology to integrated tumor taxonomy [9]. Unlike previous editions, the most significant innovation of the fifth edition was the adoption of a standardized taxonomic framework applied across the entire WHO Classification of Tumors series, rather than simply recognizing new entities. Tumors are now organized according to hierarchical levels, including site, category, family or class, type, and subtype. This approach promotes consistency across organ systems and facilitates communication among pathologists, clinicians, and researchers.

A second major innovation was the establishment of essential and desirable diagnostic criteria for each condition. This approach distinguishes between mandatory diagnostic features and supportive findings, while integrating clinical, radiologic, histologic, immunophenotypic, and molecular information into a unified diagnostic framework. Consequently, the fifth edition places a stronger emphasis on reproducibility and diagnostic standardization than any previous WHO classification [9].

The current classification reflects the maturation of molecular pathology in the field of head and neck oncology. Recurrent genetic alterations are increasingly incorporated into tumor definitions, diagnostic algorithms, and differential diagnoses. This reflects a shift from morphology-based classification to biologically informed disease taxonomy. Although morphology remains essential, molecular findings have become integral to routine diagnostic practices for several tumor groups [9].

**Fig. 1:**
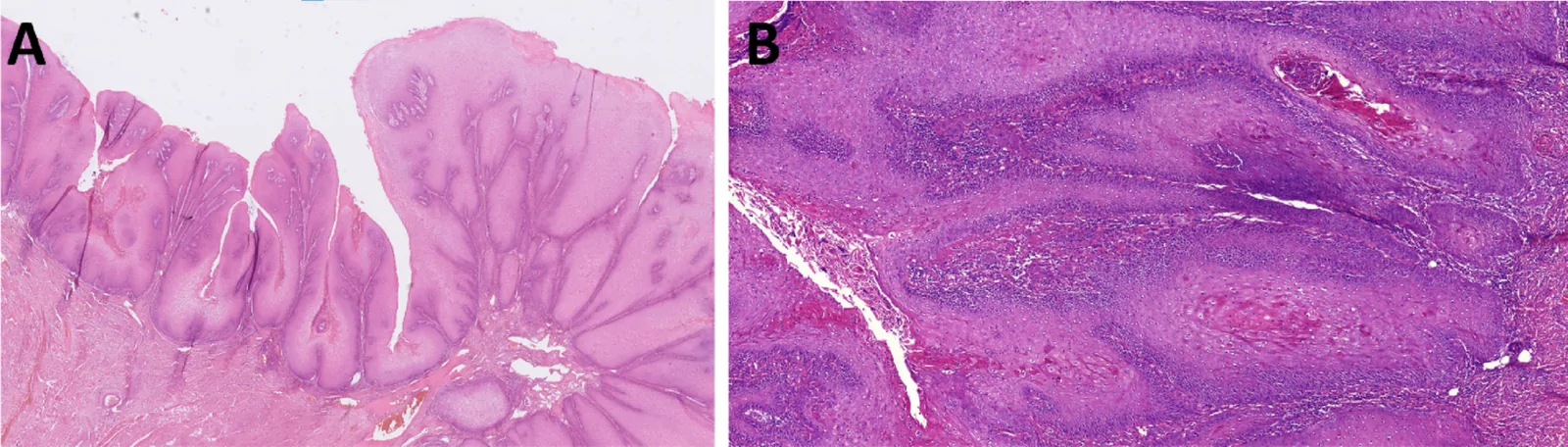
**A** Verrucous carcinoma showing a broad exophytic, verruciform proliferation of well-differentiated squamous epithelium with bulbous rete ridges and a pushing interface. **B** Carcinoma cuniculatum displaying deeply infiltrative, branching keratin-filled epithelial channels and sinuses extending through the underlying stroma (H&E)

In the oral cavity, hypopharynx, larynx, trachea, and parapharyngeal space, the fifth edition, there was a refinement in the organization. Oral potentially malignant disorders and oral epithelial dysplasia were separated more clearly, and submucous fibrosis and HPV-associated dysplasia were given dedicated discussion. The grading of oral epithelial dysplasia remains contentious, but the fifth edition expands architectural and cytologic criteria, including a clearer distinction between generalized premature keratinization and single-cell keratinization [42]. Verrucous carcinoma **(**Fig. 1A**)** and carcinoma cuniculatum **(**Fig. 1B**)** are now discussed in dedicated sections, acknowledging their distinctive clinicopathologic behavior and the oral cavity as their most common head and neck site [43].

Neuroendocrine neoplasms are another major example of taxonomic harmonization in the fifth edition [9]. Following the IARC/WHO framework developed across organ systems, the 2022 classification reserved the term “neuroendocrine carcinoma” (NEC) for poorly differentiated epithelial neoplasms and introduced “neuroendocrine tumor” (NET) for well-differentiated neoplasms [9, 44]. NETs are further categorized as G1, G2, or G3 based on morphologic differentiation, mitotic activity, necrosis, and the Ki-67 proliferative index. Poorly differentiated tumors, on the other hand, are classified as small cell or large cell NECs [9, 45]. This shift in terminology from well-, moderately, and poorly differentiated neuroendocrine carcinomas to a taxonomy that integrates differentiation and proliferative biology across anatomical sites is an important conceptual change.

**Fig. 2:**
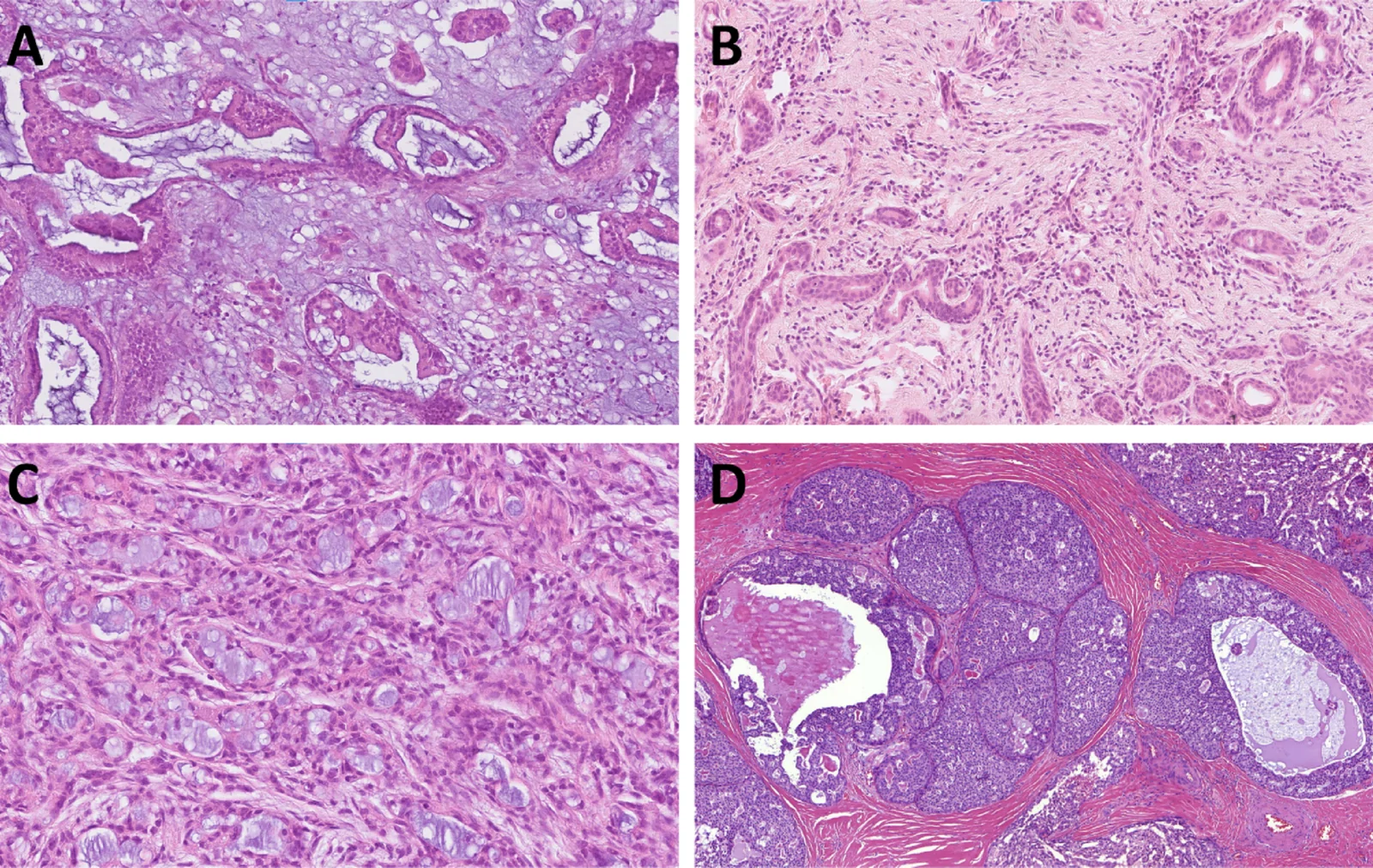
**A** Mucinous adenocarcinoma showing irregular glandular structures and epithelial clusters suspended within abundant extracellular mucin. **B** Sclerosing microcystic adenocarcinoma composed of small infiltrative ducts and cords embedded in densely sclerotic stroma. **C** Microsecretory adenocarcinoma displaying infiltrative cords and microcystic structures containing eosinophilic secretions within a fibromyxoid stroma. **D** Intraductal carcinoma, intercalated duct variant, characterized by well-circumscribed intraductal epithelial proliferations with solid and cribriform architecture within dilated ductal spaces (H&E)

The salivary gland chapter underwent some of the most substantial changes. Under the influence of advances in molecular pathology, several entities, such as sclerosing polycystic adenoma, were reclassified as tumors instead of being included among non-neoplastic proliferations [46–50]. In the same way, new malignant entities were characterized based on their molecular profiles, including mucinous adenocarcinoma **(**Fig. 2A**)**, sclerosing microcystic adenocarcinoma **(**Fig. 2B**)**, microsecretory adenocarcinoma **(**Fig. 2C**)**, and the intercalated duct variant of intraductal carcinoma **(**Fig. 2D**)** [51]. Poorly differentiated carcinomas and oncocytic carcinomas are now discussed under salivary carcinoma, NOS and emerging entities, reflecting the cautious recognition that some tumors remain in a biologically unresolved category [49]. Interestingly, the chapter was made more selective: mesenchymal tumors non-specific to the salivary glands were removed from detailed salivary classification and addressed elsewhere [49].

In odontogenic and maxillofacial bone tumors, the fifth edition maintained the broad logic of 2017 but introduced important refinements. Surgical ciliated cyst was added to the jaw cyst group, despite not being a true odontogenic cyst, because of its occurrence in the jaws after surgery, grafting, or trauma. Adenoid ameloblastoma was recognized as a new benign epithelial odontogenic tumor, important because of its overlap with conventional ameloblastoma, adenomatoid odontogenic tumor, and dentinogenic ghost cell tumor. Segmental odontomaxillary dysplasia was introduced among fibro-osseous tumors and dysplasias, while rhabdomyosarcoma with *TFCP2* rearrangement was added to malignant jawbone tumors, reflecting the growing role of molecularly defined entities [52, 53].

**Fig. 3:**
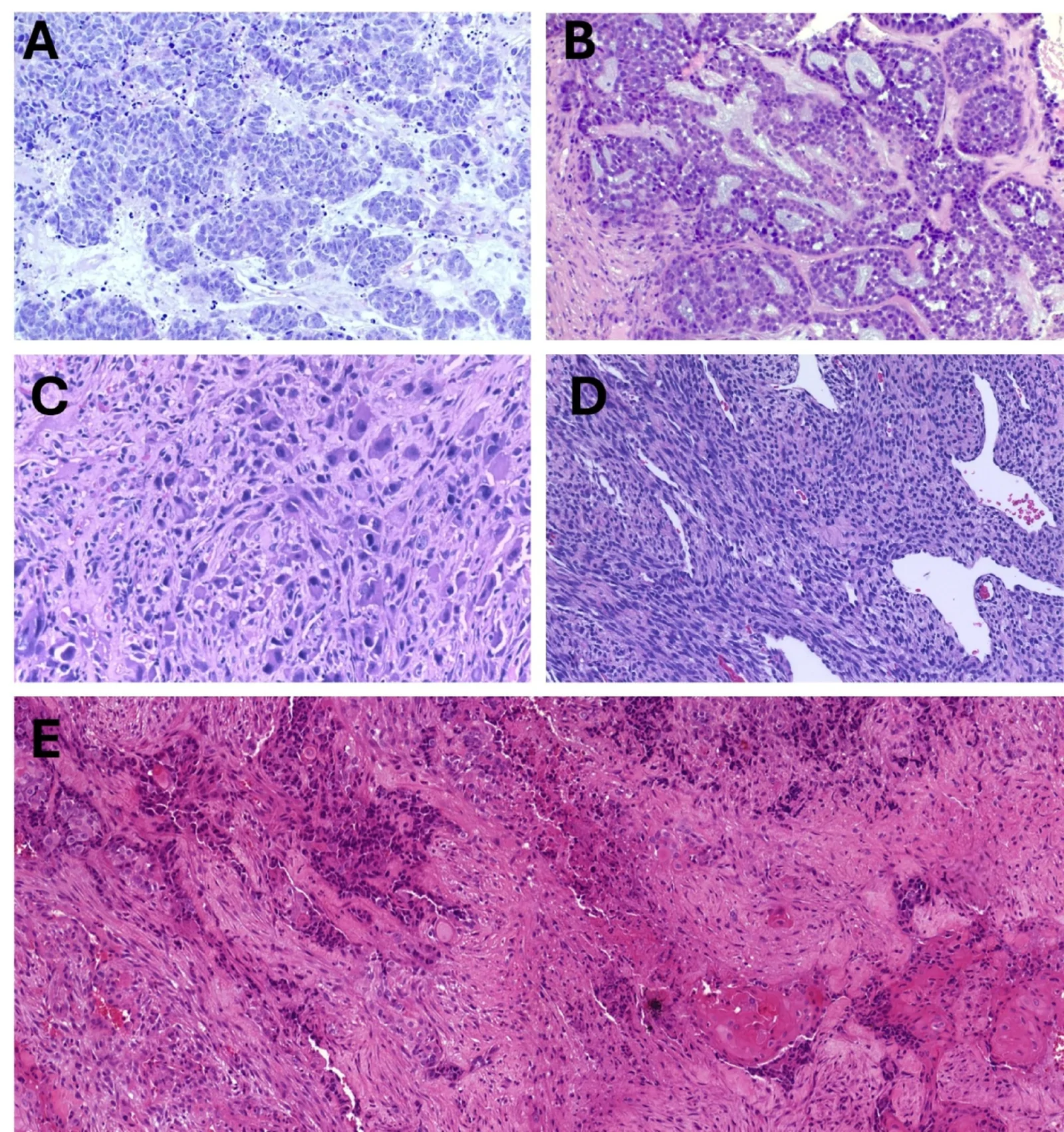
**A** Sinonasal undifferentiated carcinoma with nested architecture. **B** HPV-related multiphenotypic sinonasal carcinoma consisting of solid and cribriform nests, with interspersed myxohyaline stroma. **C** Rhabdomyosarcoma with TFCP2 rearrangement showing high-grade atypical spindle and epithelioid cells with abundant eosinophilic cytoplasm and large vesicular nuclei. **D** Biphenotypic sinonasal sarcoma consisting of a spindle cell proliferation with mild atypia forming elongated fascicles, accompanied by dilated haemangiopericytoma-like vessels. **E** NUT carcinoma arranged in infiltrative nests and cords, with foci of abrupt squamous differentiation and keratinization in a desmoplastic stroma (H&E)

Sinonasal pathology is one of the most rapidly evolving areas of the fifth edition [9, 54]. Advances in molecular diagnostics have led to the identification and refinement of several genetically defined conditions, such as SMARCB1- and SMARCA4-deficient carcinomas [55–57], biphenotypic sinonasal sarcoma [58, 59], NUT carcinoma [60, 61], and HPV-related multiphenotypic carcinoma [62, 63] **(**Fig. 3**)**. Moreover, it has been recognized that high-grade sinonasal malignancies, including mainly sinonasal undifferentiated carcinoma (SNUC) [64], but with lower frequency also neuroendocrine carcinoma, sinonasal adenocarcinoma, or olfactory neuroblastoma, are characterized by mutations of the *IDH* genes [54, 65]. Finally, *DEK*::*AFF2* squamous cell carcinoma is included as an emerging entity presently considered a subtype of non-keratinizing squamous cell carcinoma [66–68]. These tumors exemplify the growing significance of molecular alterations in defining disease entities and underscore the shift from morphology-based diagnosis to integrated molecular classification [9].

## From Classification to Clinical Impact: Challenges and Future Directions

The evolution of the WHO classification of head and neck tumors reflects more than just the recognition of new entities or changes in nomenclature. With each successive edition, the role of tumor classification has progressively transformed from a descriptive system based primarily on morphology into an integrated framework that combines histopathologic, immunophenotypic, molecular, etiologic, and clinical information. This reflects the evolution of the role of practicing pathologist. Modern diagnosis increasingly requires integrating morphologic interpretation with molecular findings, viral biomarkers, and clinicopathologic context to define biologically meaningful disease entities that directly influence prognosis, therapeutic decision-making, and eligibility for targeted treatments.

Despite these advances, important challenges remain. One of the greatest is achieving a balance between diagnostic stability and the rapid pace of scientific discovery. As novel molecular alterations and potential tumor subtypes continue to emerge, future WHO classifications will need to apply clear validation criteria before recognizing new disease entities. This approach should help preserve clinical relevance and diagnostic reproducibility while avoiding unnecessary fragmentation of rare neoplasms. Additionally, while many molecular alterations are highly characteristic, they are not entirely specific and should be interpreted alongside a careful morphologic assessment. Future classifications will likely incorporate increasingly integrated models combining genomics, epigenomics, transcriptomics, digital pathology, and artificial intelligence. Nevertheless, morphology is expected and should remain the cornerstone of diagnosis, with emerging technologies serving to refine rather than replace conventional histopathology.

## Conclusion

Over the past five decades, the WHO classification of head and neck tumors has evolved alongside advances in pathology. It has transitioned from a descriptive, morphology-based system to an integrated taxonomy that incorporates clinical, pathological, immunophenotypic, molecular, and etiological information. Each successive edition has refined diagnostic criteria, recognized new disease entities, and progressively redefined the principles underlying tumor classification. This reflects an increasingly sophisticated understanding of head and neck tumor biology. The history of the WHO classification shows that tumor taxonomy is a dynamic process driven by scientific discovery and clinical need. It is more than a historical record of changing nomenclature; it illustrates how advances in pathology continuously reshape diagnostic practice and our understanding of disease. This evolution provides the foundation for future classifications that will continue to integrate emerging biological knowledge while preserving the clarity, reproducibility, and clinical relevance that have defined the WHO classification for over five decades.

## Data Availability

No datasets were generated or analysed during the current study.

## Code Availability

No software application or custom code has been used in the preparation of this article.
